# Blockchain-Based Decentralized Cloud Solutions for Data Transfer

**DOI:** 10.1155/2022/8209854

**Published:** 2022-05-30

**Authors:** Rajit Nair, Syed Nasrullah Zafrullah, P. Vinayasree, Prabhdeep Singh, Musaddak Maher Abdul Zahra, Tripti Sharma, Fardin Ahmadi

**Affiliations:** ^1^School of Computing Science and Engineering, VIT Bhopal University, Bhopal, India; ^2^Department of Information Systems, College of Computer Engineering & Sciences, Prince Sattam Bin Abdulaziz University, Al-Kharj 11942, Saudi Arabia; ^3^Department of Computer Science & Engineering, Anurag University, Venkatapur, Ghatkesar Rd, Hyderabad, Telangana 500088, India; ^4^Department of Computer Science & Engineering, Graphic Era Deemed to be University, Dehradun, Uttarakhand, India; ^5^Computer Techniques Engineering Department, Al-Mustaqbal University College, Hillah 51001, Iraq; ^6^Electrical Engineering Department, College of Engineering, University of Babylon, Hillah, Babil, Iraq; ^7^IT Department, Maharaja Surajmal Institute of Technology, New Delhi 110058, India; ^8^Lecturer of Computer Science Faculty, Rana University, Kabul, Afghanistan

## Abstract

Cloud computing has increased its service area and user experience above traditional platforms through virtualization and resource integration, resulting in substantial economic and societal advantages. Cloud computing is experiencing a significant security and trust dilemma, requiring a trust-enabled transaction environment. The typical cloud trust model is centralized, resulting in high maintenance costs, network congestion, and even single-point failure. Also, due to a lack of openness and traceability, trust rating findings are not universally acknowledged. “Blockchain is a novel, decentralised computing system. Its unique operational principles and record traceability assure the transaction data's integrity, undeniability, and security. So, blockchain is ideal for building a distributed and decentralised trust infrastructure. This study addresses the difficulty of transferring data and related permission policies from the cloud to the distributed file systems (DFS). Our aims include moving the data files from the cloud to the distributed file system and developing a cloud policy. This study addresses the difficulty of transferring data and related permission policies from the cloud to the DFS. In DFS, no node is given the privilege, and storage of all the data is dependent on content-addressing. The data files are moved from Amazon S3 buckets to the interplanetary file system (IPFS). In DFS, no node is given the privilege, and storage of all the data is dependent on content-addressing.

## 1. Introduction

Cloud computing has achieved substantial appeal with the expansion of communication and information technologies. AWS, Microsoft Azure, and Google Cloud Platform (GCP) are three cloud computing platforms. On-demand network access to a shared pool of computer resources includes storage, networking, computing, and security. A lot of firms employ cloud computing to store a significant amount of data remotely instead of maintaining it on local equipment. The services supplied by the cloud demand extensive bandwidth and high-speed Internet, which restricts their adoption by many end-users. Similarly, vendor lock-in is a problem with cloud computing, and moving data across cloud services is challenging. Recently, decentralised storage technology has been established for storing data safely without third-party aid. One of its applications is the DFS, which stores data chunks on multiple peers across the network. Other implementations include IPFS, SWARM, and SIA. The interplanetary file system is a peer-to-peer network that stores large amounts of data without relying on central servers. IPFS leverages the notion of storing data based on content-based-addressing. It operates by breaking data into fixed-size pieces, distributing them throughout the whole network, and then generating a hash table. Current cloud customers can now store data locally, giving them more control over the data.

Security is a serious worry for sensitive and private data. Authorization or access control policies allow you to specify who has access to which resources based on certain attributes or roles. Amazon, for example, provides an Identity and Access Management (IAM) service to establish permission policies. Migrating private or sensitive data from the cloud to the DFS is not practicable until we can move the authorization policies, connected with the data on the cloud, to the DFS. Because current DFS implementations like IPFS and Sia lack permission policy definition methods, data cannot be moved from the cloud. The blockchain is another decentralised storage system that stores data in a sequence of blocks connected by cryptographic hashing of previous blocks. To our knowledge, no solution exists for transferring data and authorization policies from the cloud to DFS. We have mapped our recommended technique to move data from AWS S3 to the IPFS, and the resource-based permission policies given at AWS are added to a bespoke blockchain solution. Specifically, our contributions include the following.

*Content Migration*. In this way, data objects are moved from Amazon S3 to the interplanetary file system. Different kinds of data are saved in the cloud, transmitted to IPFS, and disseminated across the full IPFS network.

*Custom Blockchain*. The authors have constructed a customised blockchain for storing authorization policies connected with the data being moved.

*Authorization Policies on the Chain*. The authors have designed a customised blockchain for storing authorization policies connected with the data being moved. The suggested technique moves the access control policies, for instance, connected with S3 buckets. Amazon S3 is an object storage service that stores data as objects within buckets. To begin, create a bucket and choose an AWS region to access our bespoke blockchain system. These access policies are stored as blocks in an immutable ledger.

We proposed and built a custom DFS client that displays the files to the user. It leverages the regulations from the blockchain and data kept at the DFS to list and regulate access. *Implementation*. The authors have supplied implementation details to justify the feasibility of our method.

## 2. Background and Related Work

This study addresses the difficulty of transferring data and related permission policies from the cloud to distributed file systems. To the authors' knowledge, no technique exists in the literature to address data and policy transfer from the cloud to the DFS. In this section, we will give a quick overview of the subject and look at some of the best ways to deal with some of its parts.

### 2.1. Cloud Computing

Cloud computing usage is steadily increasing. On-demand dynamic and elastic resource provisioning makes cloud computing appealing to users. Using the Internet, cloud customers may access services from anywhere and on any device. The resources given by the cloud include storage, networking, computing, security, and others. The growth of the cloud also arises from the advantage of decreasing hardware and software expenses. It also decreases maintenance expenses, since the company does not need to maintain software and hardware. All programmes are operated on cloud servers and must be maintained by the providers. There are three types of cloud computing service models: infrastructure, platform, and software (SaaS). The three cloud computing platforms are Amazon Web Services (AWS), Microsoft Azure, and Google Cloud Platform (GCP) [1].

AWS is a renowned cloud provider with data centres located all over the world. In our work [2], Amazon Simple Storage Service (S3) and Amazon Identity and Access Management (IAM) are employed [3]. Amazon S3 enables scalable data backup and collection, as well as storage for analytics. Buckets are used to organise data and manage access to it. AWS IAM controls permissions and authentication for AWS services. Using IAM, administrators may establish users and groups and enable (or refuse) those individuals or groups to access resources. The IAM process involves a principal that is used as a role or an application that performs actions on AWS resources.

  Authentication: It includes authenticating the principal's identity when trying to access an AWS product.
  Request: A principal requests access to AWS resources.
  Authorization: IAM grants access only if part of the request and policy match.
  Actions: It is used to view, delete, or edit a resource.
  Resources: They are a collection of actions that are performed on resources connected to AWS.

IAM comprises several components, like users, groups, policies, and others. A user is a person who wishes to acquire access to the resources of AWS. Each IAM user is connected to one account alone. Similarly, the collection of users constitutes the IAM group. Managing groups is straightforward, since the owner may specify the rights for the group, and this permission is automatically applied to all the members of the group. IAM policies set authorization and regulate access. It is saved in the form of JSON documents. For example, a policy might enable a certain user to access the bucket of Amazon S3. The policies contain the following information:

  Who may access resources?
  What action must be taken?
  Which AWS resources may the user access?

In IAM, roles are also significant, since they are a collection of permissions that dictate what actions are allowed and refused. It is like a person who can be accessed by anyone, like an individual or an AWS service.

Economic savings are the fundamental motivator for the company to transition to cloud services. However, the security risk is a huge worry. According to the current situation, cloud computing security is the most difficult task for any company. The biggest security risk is leakage of data or the possibility of undesired access by certain unauthorised parties, stemming from insufficient data management by cloud providers or inadequate access restrictions. Similarly, the lack of compatibility between multiple cloud servers following different storing data standards is also a big concern. As a result, it is difficult for clients to transition from one cloud provider to another.

### 2.2. Blockchain

Satoshi Nakamoto's 2008 invention, bitcoin, solidified the blockchain concept with a hundred millionth of a bitcoin. Bitcoins are classified into two types: 1000 mBTC and 1000 microBTC, as well as 100,000,000 Satoshi (S). At the moment, one Satoshi is worth 0.0001332797 USD. The blockchain is a secure data system for storing data. In a blockchain network, all peers share the same copy of a database called the ledger. No central authority controls everything, and no one node grants privileges. Nodes agree to add blocks to a network, forming a chain. Every new blockchain transaction is added to the peer's ledger. “DLT” stands for Distributed Ledger Technology (DLT) [3]. To add blocks to a chain, bitcoin uses PoW consensus. Each network node calculates a hash value. The criterion is to compute a value equal to or less than the input value. When a node achieves a goal value, it is broadcast to the whole network. It is added to the blockchain if more nodes approve it. Mining is the process of computing the hash. In the PoS consensus method, miners must own a certain amount of money [4]. Since people with greater money might attack a network, many solutions to proof of stake consensus are given, such as DPOS (Delegated Proof of Stake). Depending on the application, blockchain solutions might be permissioned or permissionless.

The permissionless blockchain is public. No body has ultimate control over the network. This functionality helps to have secure and immutable data. The blockchain's authority is evenly distributed across nodes, making it completely scattered. Cryptocurrencies like bitcoin, Litecoin, and Ethereum heavily use it. Ethereum, created by Vitalik Buterin, is the most popular permissionless blockchain technology, an open-source distributed computing platform for DAap development.

The main difference between bitcoin and Ethereum is that Ethereum is focused on running computer programmes called smart contracts. Instead of bitcoins, Ethereum miners strive to earn ether, the network's own coinage. Similarly, gas must be paid for every network action. The gas limit is the maximum amount of gas that the user is willing to spend on each transaction [5].

Hyperledger Fabric is one example of a permissioned blockchain. It is a platform that delivers a distributed ledger solution with great flexibility, scalability, and privacy. The Linux Foundation launched the Hyperledger project in 2015 to spread blockchain technology. It features a ledger, smart contracts, and a way for participants to interact. It allows a group of parties to construct a ledger for a transaction. In a network, some competitors might not desire every transaction. In a Hyperledger Fabric smart contract, it supports Go, Node, and others. Consortia blockchain has various degrees of authorization. The fabric conserves resources. It has fewer nodes than a public chain and works in parallel. It has two virtual nodes: Endorsers and Consensus Nodes. A validator of transactions and chain codes confirms a previously verified transaction. Compared to bitcoin, it allows for greater network division of labour.

### 2.3. Distributed File Systems

A distributed file system is a file system spanning numerous nodes and maybe multiple locations. It lets users exchange data and storage resources by using a common file system. Accessing the file relies on a client/server architecture. A fundamental aspect of DFS is the high availability of information, since it continues its operation after failure as data is duplicated on several nodes. DFS also refers to transparency by not telling a user about things that are not important, like replication transparency, so a user does not know that there are copies of data.

Swarm is an Ethereum-based content distribution service, and data access on a network is location-independent. With a strong connection to Ethereum, it has the benefits of both smart contracts and Filecoin. Two key elements that set the Swarm apart from other DFSs are the “upload and disappear” (upload material and permit to go offline) and its incentive structure. The service of delivering pieces is paid, and nodes can exchange resources. To encourage the nodes, Swarm employs SWAP (Swarm Accounting Protocol) [6]. In Swarm, nodes keep data chunks and profit for sale when some retrieved request is received; otherwise, the request is transferred to the next neighbour node. The main purpose of Swarm is to offer an infrastructure for creating dApps.

#### 2.3.1. Storj

Storj is a decentralised platform that preserves data without third-party services [7]. It is a client-side encrypted peer-to-peer network. Initially, the file is separated into smaller bits, and the distributed hash table is built, where all information regarding shards is maintained. The file's uploader is the only individual who has access to shards of its original file as the data owner's key encrypts the hash table. In the network, farmers who offer their free drives to store those shards of files are utilised. They earn a micropayment for keeping and maintaining a file. Therefore, they are rewarded for remaining active in the network.

#### 2.3.2. Sia

It is a blockchain-based cloud storage solution that works without a third party. Peers in Sia may rent their hard drives and collect incentives. The two primary components of Sia's network are renters and the host. The hosts rent their storage facility to renters by publicising their storage resources. Hosts also have the power to turn away renters if the information they give is illegal or too private.

#### 2.3.3. IPFS (Interplanetary File System)

It is a peer-to-peer version-controlled file system [8] in which files are kept in the form of content-based-addressing. It is one of the most common solutions, and we will discuss it in the next subsection.

### 2.4. The Interplanetary File System

It is a peer-to-peer file system where files are stored using content-based-addressing [8]. It combines the Distributed Hash Table, Bitswap (from BitTorrent), and Merkle DAG (from Git Protocol) (inspired by Git Protocol). IPFS is designed to replace HTTP, since large files cannot be transmitted over HTTP. Also, client server is used, resulting in low latency; real-time streaming is impossible with HTTP. IPFS addresses all these issues. Unlike HTTP, IPFS preserves data based on the content addressed. When data is posted to the IPFS network, it generates a hash that is used to request data. It pays storage providers with cryptotokens. Copies of the data are made and distributed over the network for backup. When a user requests data, it looks for the closest copy, which increases availability and reduces bottlenecks. IPFS has the following features.

A Distributed Hash Table is a type of hash table that is used to store data across network nodes. It is like a hash table. Using DHT, any network node can request a file, video, or other items.

It uses BitTorrent (Bitswap) technology to exchange data over a network. It is a peer-to-peer file system for untrusted swarms. The Bitswap mechanism distinguishes IPFS from other DFS.

Merkle DAG is IPFS's strongest feature due to content-based-addressing storage and tamper resistance. IPFS uses hashes to reference data blocks and objects in a DAG. It uses a Merkle Tree or Merkle DAG Tree, like Git Version Control. A file larger than the block size is broken into sections, saved as hashes on peers, and the Merkle DAG Tree is generated. It also records all file versions on the network in a scattered manner.

**Figure 1:**
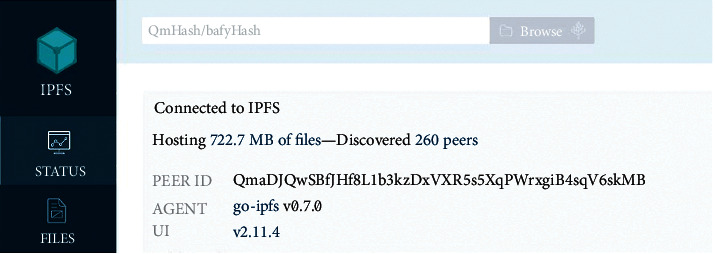
Unique Node ID in the form of a hash.

Like in Figure 1, each IPFS node has its own Node ID, which is a hash of the public key. They save data locally and get prizes. Each node has a DHT that keeps track of other nodes and their data. Users on a local network can engage with each other even if the Internet is offline. No server is required, so it is completely scattered, lowering network costs.

*Content-Addressing*. The hash refers to IPFS objects. If the user wants any file, then the IPFS server will ask for the hash matching the file [9]. It employs content-addressing at the HTTP layer. All hashes in IPFS utilise base 58 and start with “Qm.”

*Versioned File Systems*. IPFS describes the data structure by utilising Git technology, which regulates file system versions. It utilised the attribute “commit,” which points to the file system with names like “parent0,” “parent1,” and so forth.

*DHT (Distributed Hash Table)*. IPFS is a cross between DHT and SFS (self-certifying file system). This data storage is more scalable and decentralised than the cloud. Using a self-certifying file system also eliminates the need for authorization [10]. Like the web, IPFS works by uploading a file to acquire a unique cryptographic hash string. The hash string also works as a URL on the web. Filecoin is an IPFS reward.

*Authentication*. The authorization or access control mechanism restricts resource access. The authorization procedure follows the authentication process, which verifies the claimant's identity. It has a password. Authorization procedures, on the other hand, use policies to determine who may access what resources, when, and why. This includes a subject, an object, and an action. UBAC, ABAC, and RBAC are all types of access control.

This paradigm is based on the organization's duties. The administrator defines a role's duties and access privileges. This access control allows one person to play many roles. RBAC is simple to implement. Many businesses use this, since RBAC does not need to be changed when employees leave or change roles. Similarly, new employees may gain access quickly. Aside from scalability and role explosion, RBAC offers several advantages [11].

*ABAC (Attribute-Based Access Control)*. The property might be user, environmental, or resource. User attributes include name, role, organization, and ID. Access time, location, and other environmental factors may be considered. Resource attributes include resource owner and filename. ABAC is a broader model, since it includes roles as an attribute [12].

The authorization domain has been a very active and well-researched research topic, with several solutions to tackle the diverse difficulties related to authorization. In this work, we will focus on approaches that leverage blockchain for access control or a DFS implementation like IPFS. The technique proposed in [13] combines IPFS, Ethereum, and attribute-based encryption to provide fine-grained data access control. According to the authors, no third-party key generator is required. Owners of data can enable fine-grained access control by encrypting data using secret keys and access policies. This paper proposes a smart contract-based strategy to protect personal data from untrusted sources [14]. Their work relied on trust from several nodes to facilitate data access. The recommended strategy contrasted two possible alternatives: Secret Sharing and Threshold Proxy Reencryption. Unlike our method, theirs relies on attribute-based encryption rather than authorization components. The Ethereum-enabled IPFS version is used and creates an access control list smart contract that IPFS software enforces [15]. When a user uploads a file, it gets split up by IPFS. For the smart contract, these pieces have a content identifier (CID). The permission storage additionally checks the transaction against the CID to make sure it is not empty or has the same owner. When the file is properly verified, control returns to IPFS. If someone wishes to obtain a data file, the request is sent to the data owner, who uses smart contracts to grant or deny access. Their approach does not cover data transmission from the cloud and the accompanying permission restrictions. Their proposed authorization paradigm is not expressive enough to reflect cloud authorization standards like AWS IAM. It is better to use a custom-built blockchain instead of Ethereum because it gives us more control and does not need to pay transaction fees.

Many researchers advocate using smart contracts on the blockchain to formalise access control restrictions. They show smart contracts as resources to be safeguarded. ABAC is specified in XACML using PEP, PAP, and AMs. The authors want the resource owner to be unable to restrict access without leaving an auditable record. The article describes a modular consortium for IoT and blockchain network design for decentralised access to provide IoT users control over their data. The article develops a software stack of smart contracts using IPFS [16]. The RBAC-SC framework defines access policies. Their logic is that the user can only access data related to their work. The user-role listings are built using a smart contract. The Ethereum architecture is used to secure user data access [17]. They deployed two types of smart contracts: a policy contract that lets the data owner specify how much data to send to each requestor and a data access smart contract. The user stores the data on the blockchain and uploads it to IPFS. The requestor does not create a website but instead connects with the data owner. The data owner sends the details to the requestor based on the user's authorization. Data sharing systems depend upon a trusted third party (TTP) which lacks transparency and security. To tackle this issue, [18] has developed a solution based on blockchain, IPFS, and encryption. The suggested approach achieves security and authenticity for the owner by employing smart contacts. Likewise, the DFS and Distributed Ledger Technologies (DLT) capabilities are employed for the construction of a decentralised personal information management system. For real-world assessment, they built an Intelligent Transportation System use case. A novel strategy is provided based on IPFS and Hyperledger technology, which may enable audit access to files as it reveals who has downloaded them, thus providing evidence for both dispute resolution and forensics [19]. Another technique is presented which enables patients to regulate the sharing of their health data by employing an attribute-based encryption scheme in a distributed file system. They illustrate that, together with privacy protection, it also gives the capacity for data secrecy [20]. The proposed solution can also be compared with Smart Vault, a platform that allows users to exchange files with a predetermined group of individuals. A smart contract manages the access control list. Their approach does not cover data transmission from the cloud and the accompanying permission restrictions. Their proposed authorization paradigm is not expressive enough to reflect cloud authorization standards like AWS IAM. There are no transaction fees if we use a custom-built blockchain instead of Ethereum [21].

A cloud user might have various access privileges to the same resource, ACaaS for public IaaS clouds [22]. The architecture manages many access control policies and models. We use a unique EHR sharing architecture that combines aspects of blockchain and IPFS for mobile cloud computing [23]. They created an access control system to ensure EHR exchange between patients and doctors (patients and medical providers). They used mobile Android apps and AWS to evaluate security factors such as avoiding single points of failure, availability, and integrity. That method can identify and prevent unauthorised access to E-Health data. To overcome the problem of multiple authorizations in E-health, they use blockchain technology. The patient has the right to only share personal data with trusted people in their approved way.

For an incentive-based blockchain-based access control solution for e-health systems, in their view, a patient has the right to share their records. The incentive system also encourages the active sharing of medical information. Their main interest is fine-grained sign-up access control.

A decentralised storage system uses blockchain technology and a private keyword search approach. There is a blockchain-based solution for a private keyword search strategy in a decentralised storage system [24]. The proposed approach is still theoretical, and its stability and feasibility have not been demonstrated. There is an IoT-based method for building distributed and trustworthy access control policies They made a lot of access contracts (ACCs) on the Ethereum blockchain to make sure people and things could get in and out of each other and things.

### 2.5. Blockchain Advanced Machine Learning

Large amounts of data are necessary to develop excellent machine learning models. Large data raises the total throughput, which aids in drawing broader conclusions and is more efficient and dependable. One of the reasons why big data is important in machine learning is overstated [25]. However, blockchain databases that have shared data in machine learning have improved machine learning models and safer data. For data exchange, the decentralised nature of blockchains allows for data sharing across nodes. This facilitates data access for linked machine learning models. Data collection has been the basis of most machine learning research works. Previously, researchers had to fight to gain a set amount of data for investigation [26–28]. This issue not only led to less dependable and inefficient models but also hampered several research projects. With big data, this barrier may be overcome. A trustworthy person would be involved to gain an adequate amount of data. Trustees would then be compensated for the data collected. Because of decentralised data sharing, databases may be able to give data to researchers for big research projects without a trusted third party.

Data from decentralised data sources provides more and safer data from both internal and external sources. In sources of intrinsic data for local and metropolitan areas, the data from a certain company's branch might be stated to be local [29, 30]. Combined data from different companies' metropolitan data is represented by branches. Using a machine learning model instead of simple local data is more efficient. Extrinsic data is exchanged with data from connected firms. When forecasted, machine learning models cannot produce better forecasts. Aside from obtaining massive amounts of data at virtually no cost, it is also as safe as heaven [31]. People who use machine learning to look at a lot of secure data end up with better machine learning models for things like forecasting, illness detection, voice and speech recognition, face detection, and more, to name a few.

This research tackles the issue of moving data from the cloud to DFS. To our knowledge, there is no way to move data and policies from the cloud to the DFS.

**Figure 2:**
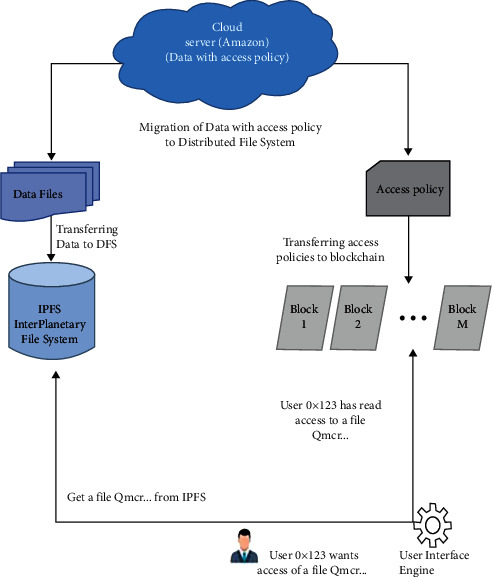
Different components of the suggested technique.

Figure 2 shows the different components of the suggested technique.

## 3. Proposed Approach

This study addresses the difficulty of transferring data, and related permission policies, from the cloud to the DFS. Different components of the suggested strategy are outlined in Figure 2. Our aims include moving the data files from the cloud to the distributed file system. In DFS, no node is given the privilege, and storage of all the data is dependent on content-addressing. Furthermore, all files saved in the DFS should be protected against unauthorised access, and only a subject with the required characteristics should have access to any part of the data. This may be done by installing role-based or user-based access control on the data, as provided by major cloud providers. The two main parts of the suggested solution are moving data to the DFS and putting access controls on the data that has been moved.

To further describe the components of the proposed strategy, we may map them to the current DFS and the cloud provider. For this reason, we have chosen AWS as the cloud provider and the interplanetary file system (IPFS) as the DFS, as indicated in Figure 2. The data files are therefore moved from the Amazon S3 buckets to the IPFS, and the authorization policies associated with the S3 buckets, the resource-based IAM rules, are then utilised for implementing the access control on the IPFS files. The proposed technique may be used by other cloud providers and DFS as well.

### 3.1. Transferring Data from the Cloud

Amazon Simple Storage Service (S3) and Amazon Identity and Access Management (IAM) are employed in our work. AWS IAM manages the permission and authentication components for the services supplied by AWS. Using IAM, administrators can set up users and groups and let them access resources or block them from doing so.

**Figure 3:**
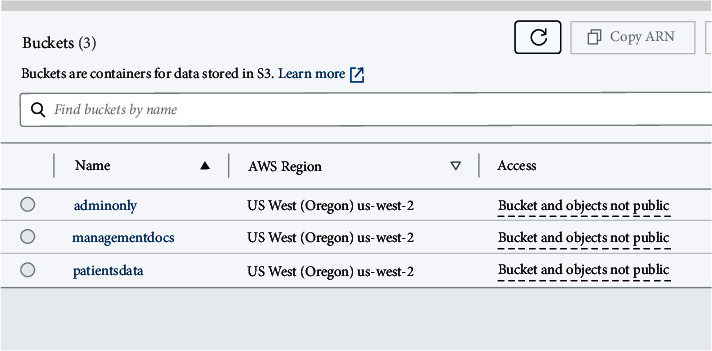
AWS S3 buckets.

Amazon S3 provides a scalable storage location for data backup, collection, and analytics. The data is grouped into units called buckets, and access controls may be linked to the buckets. An object consists of any file and metadata that describes the file. To store an item on Amazon S3, the user uploads the files in buckets, as illustrated in Figure 3.

Scalability, data availability, security, and speed are unrivalled in Amazon S3. Data lakes, cloud-native applications, and mobile apps may be stored for clients of various sizes and sectors. To fulfil particular business, organisational, and regulatory needs, you may save expenses, organise data, and implement access restrictions.

Buckets are like folders in that they allow users to store a variety of files. Users are required to provide the bucket name to access their data. Once the bucket's name has been established, it cannot be modified. A user can also pick a bucket's region in AWS. Bucket regions are places in the world where AWS has servers that are physically separate from each other so that data can move faster.

As a first step, object files (the actual content) should be transferred from S3 buckets to IPFS as a first step in the suggested strategy (interplanetary file system). Versioning management is provided through IPFS, a peer-to-peer technology. Using content-addressing, it saves information. There is a default chunk size of 256 kb in IPFS, so when files are moved from Amazon S3 to IPFS, they are dispersed throughout the whole network. The unique chunk ID is used to identify and store each chunk on distinct peers. DHTs keep track of the chunk IDs and the peers that store them, so that the chunks may be retrieved at any time. Using the Distributed Hash Table (DHT), a user may request a file and have it sent to the appropriate node. Using Qm, a user can gain access to a file via the root hash. Data duplication is not possible, since each file is saved with a unique hash. In addition, users can attach important files to their nodes.

**Figure 4:**
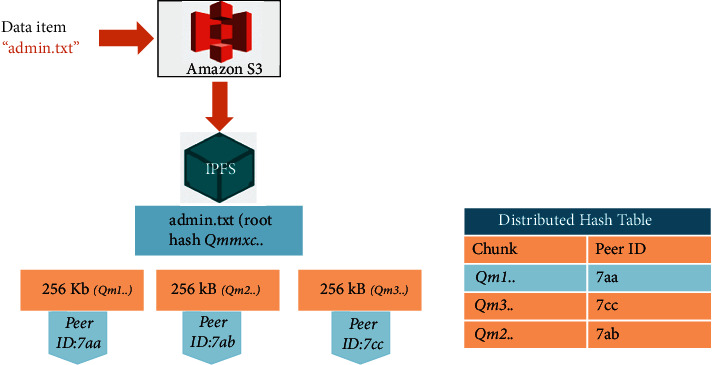
AWS S3 to IPFS migration.

To move all of Amazon S3's buckets to IPFS, we utilised Go routines. For instance, the Amazon bucket contains the file admin.txt. The admin.txt file is split into 256 kb pieces when uploaded to IPFS through the API. The content of each chunk is represented by a hash code. The Merkle DAG, a hierarchical data structure composed of CIDs for all chunks, is the result of this process. Additionally, IPFS maintains a Distributed Hash Table against the peer ID information used to store file chunks, as seen in Figure 4.

The root hash of the admin.txt is now required for any user who wants to access it. Using its DHT, the IPFS network discovers which peers have saved chunks and content of the file admin.txt. IPFS verifies the file's presence in Merkle DAG Trees.

### 3.2. Policy Based on the Cloud

AWS IAM is responsible for the authentication and authorization of AWS services. Users and groups may be created, and administrators can grant or refuse access to resources using IAM's authorization features. As part of the IAM process, you will see the following:

  There is a role or application that uses an AWS principal to conduct operations on resources.
  During the authentication procedure, an AWS service provider checks the principal's identity.
  A principal requests access to AWS resources by submitting a request.
  Only if a portion of the request and policy match can IAM grant access.
  It is useful for viewing, deleting, or editing a resource.
  An AWS resource's set of activities are called a resource's resources.

Amazon establishes policies and associates them with IAM accounts or resources. Each of them is an IAM identity. An IAM principal submits an AWS request, and the policy is implemented. To limit who has access to certain data, Amazon provides a variety of restrictions. The following are a few of the regulations:

Policies that authorise access based on a user's name, group, or position are known as identity-based policies.

Resources are the focus of resource-based policy. For example, S3 buckets can be assigned a resource-based policy. Permissions are granted in accordance with the principal.

In contrast to resource-based policies, access control lists (ACLs) are the sole policy type that does not accept JSON. The IAM principal is used to grant access via cross-account authorization.

**Figure 5:**
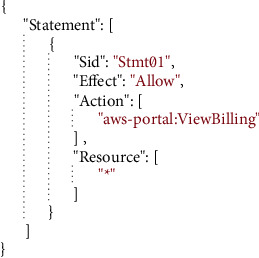
A typical JSON document structure supported by Amazon's access policies.

The following are some of the components of an effective access policy:

  It is the primary policy container that holds many aspects. A policy can have several statements added by the user.
  It tells you whether you can access the policy.
  This gives the list of resources to which the actions are applied.
  A list of permitted and prohibited acts are provided to the principal.
  Example JSON-based AWS permission policies are shown in Figure 5.
  Cloud-based transfer of authorization policies.

We have already covered how material from the cloud may be transferred and how policies are defined there in the previous sections. However, how these regulations may be used in a decentralised system remains the most pressing issue. A decentralised technique for policy design is necessary, since the content storage is decentralised. Blockchain technology can help with this. Blockchain networks use a ledger, or a distributed database, to keep track of all transactions in the network. A chain is built by a group of nodes agreeing to contribute blocks to the network. We used a custom-built blockchain that was made for the suggested method of storing permission policies decentralised.

**Figure 6:**
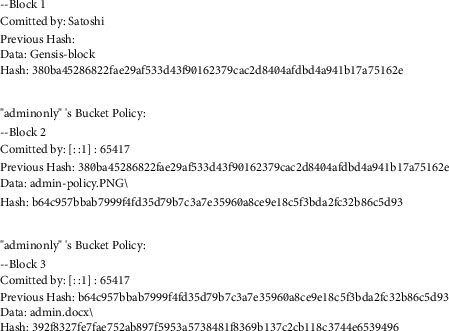
Blocks (for S3 buckets) of the custom blockchain.

**Figure 7:**
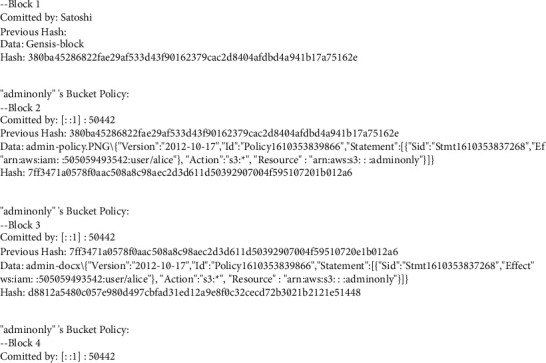
Custom blockchain blocks with authorization policies.

For joining other nodes and peers in our private blockchain, central node policies or regulations are established. An AWS login is used to set up the network and access policies are sent to our private blockchain, where Satoshi is named the central node. Policy migration creates an immutable chain of policies that are appended to blocks on the private blockchain. Figures 6 and 7 demonstrate how the preceding block's hash and an access policy designed to prevent data tampering are kept in each block. In the blockchain, the whole JSON document that represents the cloud's resource-based policy is recorded. The JSON document is eventually parsed from the blockchain when a request is made and contains information about the topic, object, and actions. Anyone who wants to be a part of the blockchain network must use their Amazon credentials to become a “node.” When a user attempts to join the network, access policies are reviewed and the blockchain is copied on each node. All the data files that the user has authorised access to are visible to him. That is how we were able to shift data from a cloud server to a decentralised network using IPFS and blockchain technologies. One of the most important aspects of our AWS rules is the principal component, which contains the username in our blockchain. Because of this rule, all privileges are awarded. Every node that joins our network gets a copy of our modified blockchain, which goes through the whole block and lets you access files based on your AWS username.

AWS credentials and an AWS username are required for anyone wishing to connect to the network in order to view the data files. His account is activated when the authentication procedure is completed and our customised blockchain is installed on his computer. All blocks in the blockchain that store access policies are connected after the authorization procedure has begun. For each folder a user requests, the blockchain traverses all blocks and checks the name of the folder against the AWS username. If the folder access policy includes the AWS username, then the root hash of all files in the folder is shown. Qm is the form used for all these hashes. A Distributed Hash Table is used by the IPFS network to get all of the chunks from other people. It then reorganises these chunks so that they can offer the file the user wants, using this root hash.

### 3.3. Details Related to the Use of This Solution

**Figure 8:**
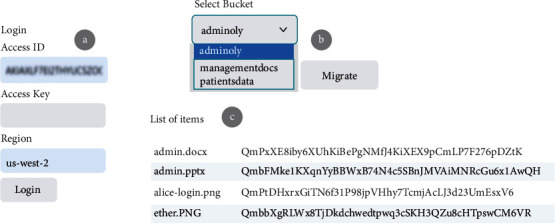
Satoshi node movement of content.

Web applications utilising Golang have been created to demonstrate the feasibility of the suggested strategy. The S3 buckets in Figure 3 are a good illustration of this point. When the Satoshi node joins the network using its AWS access ID, access key, and bucket region, as seen in Figure 8(a), the procedure gets started. One of our network's primary nodes is Satoshi. Our custom-built blockchain relies on it to move data from Amazon S3 buckets and permission policies from the cloud.

To create HTML output from the input code, our programme makes use of the Golang “text/template” package. To connect to AWS services using the AWS SDK in Golang, a session containing configuration information for the service client must be generated. Additional request handlers can be included in the session. Some fundamental packages must be imported to build sessions for bucket regions and credentials required to connect with AWS in a Golang application. To facilitate communication between our application and the Amazon Web server, we then started a new instance of the S3 client (AWS). Following a successful login, all buckets and their objects (List Objects API method) can be viewed, as shown in Figure 8(b).

After that, the user may begin the transfer from Amazon S3 buckets to the distributed file system (IPFS). Go-ipfs-api simplifies communication between IPFS and web applications because it is developed in Golang. To begin the migration process, you must first initialise your node in the IPFS network using the IPFS daemon command, which displays information such as the API server listening port and the Gateway server listening port. To contribute data to the IPFS network, we must use the “Add File” function when our node is ready. It uploads the file and generates a hash code in the form of “Qm,” as seen in Figure 8(c).

**Figure 9:**
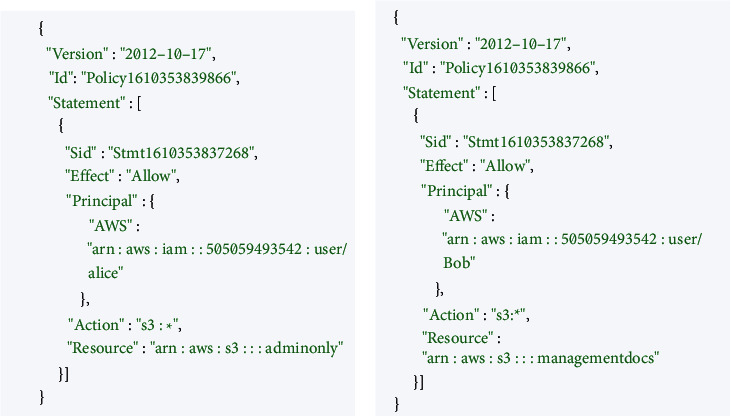
The S3 buckets' admin-only and management's authorization policies.

It is necessary to move the access control policies associated with the cloud buckets over to the custom private blockchain after the files have been put on the IPFS network. As an example, Alice can only access the admin-only S3 bucket shown in Figure 9(a), because of the Cloud's permissions policy, which you can see in the figure.

If we look at Figure 9(b), we see a user named Bob who has access solely to the management bucket. To migrate policies from the Satoshi node to our private blockchain, we must click on the policy button. To prevent data tampering, all blocks in the blockchain have access controls and are connected, as seen in Figures 6 and 7. Both the content and permission policies have been transferred from the cloud to IPFS and blockchain now. If Alice has access to the admin-only bucket, she will not be able to use her credentials to get access to the management bucket. Bob, on the other hand, can only access the bucket for management docs. Three significant trust challenges exist in cloud computing. When users upload data, code, or processes to distant cloud servers, they lose control. Transparency Cloud computing is a mysterious black box that raises concerns about privacy. There is no security guarantee. While most of the cloud service providers cite SLAs, the rationale is typically ambiguous and perplexing.

## 4. Conclusion

The use of cloud computing has been steadily increasing over time. Cloud computing is attractive to customers because of its dynamic and elastic resource provisioning. Many issues remain, including high bandwidth needs, data security, and vendor lock-in. Nonetheless, it is a promising new technology. This paper addresses the issue of migrating data from the cloud to DFS, as well as the associated authorization regulations. We do not know of any way to move data and permissions from the cloud to the DFS. We have used Amazon Web Services' content and regulations as a testbed for our approach. As a result of our strategy, data is transferred from Amazon S3 to the interstellar file system. Various kinds of data are backed up in the cloud, moved to IPFS, and then dispersed throughout the IPFS network. Access control policies, such as those associated with S3 buckets, will be transferred to our bespoke blockchain system under the suggested strategy. Our immutable ledger is made up of blocks that are linked together to store these access policies. A bespoke DFS client has been proposed and deployed by our team. To manage access, it leverages policies and data stored in the DFS.

## Data Availability

There is no dataset used in this study.
